# Heatwave increases nighttime light intensity in hyperdense cities of the Global South: a double machine learning study

**DOI:** 10.1098/rsta.2024.0568

**Published:** 2025-11-06

**Authors:** Ramit Debnath, Taran Chandel, Fengyuan Han, Ronita Bardhan

**Affiliations:** ^1^ University of Cambridge, Cambridge, UK; ^2^ University of Edinburgh, Edinburgh, UK

**Keywords:** heatwaves, nighttime light, Global South, double machine learning, causal inference, climate adaptation

## Abstract

Heatwaves, intensified by climate change and rapid urbanization, pose significant threats to urban systems, particularly in the Global South, where adaptive capacity is constrained. This study investigates the relationship between heatwaves and nighttime light (NTL) radiance, a proxy of nighttime economic activity, in four hyperdense cities: Delhi, Guangzhou, Cairo and São Paulo. We hypothesized that heatwaves increase nighttime activity. Using a double machine learning (DML) framework, we analysed data from 2013 to 2019 to quantify the impact of heatwaves on NTL while controlling for local climatic confounders. The results show a statistically significant increase in NTL radiance for Guangzhou, Cairo and São Paulo when a heatwave event lasts at least 2 days, indicating a rise in nighttime activities. However, when we extend the definition of the heatwave beyond the 2-day threshold, such an increase in the NTL values is reduced. We derive insights to improve resilience to the nighttime effects of heatwaves in urban areas.

This article is part of the theme issue ‘Urban heat spreading above and below ground’.

## 1. Introduction

Heatwaves are becoming increasingly frequent and severe, posing significant threats to urban quality of life. This trend is projected to worsen owing to climate change and rapid urban expansion, particularly in the Global South, where adaptive capacity is often limited. Understanding and mitigating the impacts of heatwaves in these regions is critical to reducing population vulnerability and improving resilience. Urban heatwaves are driven by a combination of factors, including the urban heat island (UHI) effect, which arises from human activities (e.g. transportation, air conditioning and building operations), urban infrastructure (e.g. asphalt pavements, narrow streets and high-rise buildings) and broader climatic changes such as rising global temperatures and changing atmospheric circulation patterns [1,2].

At a macro-level, heatwaves have been shown to significantly impair economic and labour productivity. For example, daytime work efficiency can decrease by up to 10% during extreme heat events [3], with particularly pronounced impacts in warmer climates. Rapid and often unplanned urbanization, a characteristic of many cities in the Global South, exacerbates heat-related discomfort, potentially triggering urban migration and increasing public health risks, including mortality [4–6]. Although cities in low-income regions face climate stresses similar to those in wealthier areas, they often lack the resources and infrastructure to mitigate these challenges effectively [4,7,8]. Consequently, this increases the vulnerability of the population to heatwave events.

Existing research, primarily focused on Europe, North America and parts of Asia, has demonstrated that heatwaves intensify the effect of UHI, particularly at night, when the temperature difference between urban and suburban areas becomes more pronounced [2,9,10]. Nighttime heatwaves also pose increased health risks, as reduced adaptive capacity during sleep can lead to increased mortality from prolonged exposure to elevated temperatures [11]. Extreme heat also negatively affects sleep quality at night and increases awake and artificially lit time [12]. In addition, there has been growing literature on exploring the economic impacts of heatwaves on an aggregated scale. For example, Babii *et al*. [13] have integrated machine learning (ML) into econometric models to improve the accuracy of economic forecasts, particularly in the context of climate-induced fluctuations [14]. Similarly, Buster *et al*. [15] developed computationally efficient ML methods to improve urban temperature estimates, which are critical to evaluating the economic benefits of heat mitigation strategies [15]. Chakraborty & Stokes [16] introduced an ML framework that adapts to city-specific nighttime light (NTL) signatures, allowing the tracking of urban changes and providing insight into economic dynamics [16]. However, there is limited evidence that economic activities are shifting towards night during heatwave events in urban areas (see §2 for a detailed review of the literature).

The primary objective of this study is to establish a robust association between heatwave events and NTL radiance in hyperdense urban areas of the Global South. To achieve this, we employ a double ML (DML) approach which combines the flexibility of non-parametric ML models with robust causal inference techniques. DML, introduced by Chernozhukov *et al*. [17], enables the estimation of causal effects while controlling for confounders and providing valid confidence intervals [17,18]. By applying DML, our aim is to establish a direct link between heatwave events and variation of NTL intensities while controlling for local climatic conditions to enable better heat-resilience planning in conditions where climate adaptive capacities are constrained.

Our analysis focuses on four hyperdense cities in the Global South: Delhi (India), Guangzhou (China), Cairo (Egypt) and São Paulo (Brazil). These cities were selected for their heterogeneous climate conditions, socio-demographic characteristics and rapid urbanization, making them ideal case studies to understand the diverse impacts of heatwaves on urban systems. NTL serves as a proxy for human activity and energy use, making it a valuable indicator of urban productivity and adaptation during extreme heat events [16]. By examining these cities, we aim to provide information on how heatwaves influence NTL in different urban contexts when confounded with heterogeneous climatic factors (see table 1). The relationship between heatwaves and NTL established in this study has significant implications for planning for heatwave adaptation in areas of limited resources. Specifically, our findings establish that heatwaves influence NTL intensity values, indicating a change in urban nighttime activities. This relationship can help policymakers and urban planners identify areas where heatwaves disproportionately disrupt urban activities, including economic productivity. This information can guide targeted heatwave interventions and implement heat-resilient urban design. Furthermore, by understanding how heatwaves alter NTL, cities can better allocate limited resources to mitigate the impacts of extreme heat, thus reducing vulnerability and enhancing resilience in the face of a warming climate.

**Table 1.** Confounding variables used in this study.

category	variables	descriptions
climate variables	cooling degree days (CDD)	a measure of how much (in °C), and for how long (in days), outside air temperature was higher than a specific base temperature.
	temp (max)	maximum recorded temperature in a day (in °C)
	humidity	the amount of moisture in the air, expressed as percentage (%)
	dew	the temperature (°C) at which air becomes saturated with moisture
	cloud cover	the fraction of the sky covered by clouds, measured in percentage (%)
	precipitation	the amount of rainfall or snowfall (in mm) recorded per day
	wind speed	average wind speed for the day measured in km h^−1^
	solar energy	total solar radiation received during the day measured in kwh m^−2^
lag variables	*CDD_Lag*{*i*}, {*i*} ∈(1, 2, 3)	CDD lagged by *i* days, representing the heat intensity from previous days to capture delayed effects
	*Humidity_Lag*{*i*}*,* {*i*} ∈ (1)	humidity from the previous day, accounting for the impact of past moisture levels
	*Tmp_Lag*{*i*}, {*i*} *∈*(1, 2)	average temperature lagged by *i* days, capturing delayed effects of temperature variations
interaction terms	heatwave—humidity	interaction term between heatwave events and humidity, capturing how humidity modifies the effect of heatwaves
	heatwave—solar energy	interaction between heatwaves and solar energy assessing how solar radiation changes during heatwave conditions
	Temp (max)—cloud cover	interaction between maximum temperature and cloud cover, measuring how cloudy days affect the impact of high temperatures

The remainder of this paper is structured as follows. In §2, we provide a detailed background on the relationship between NTL and urban productivity, current approaches to measuring productivity shifts during heatwaves, and the limitations of NTL data. In §3, we describe the data acquisition, preprocessing and modelling approaches, including the DML framework, the set-up of the treatment effect model, and the robustness checks using sensitivity analysis. In §4, we present the results and, in §5, we discuss their implications for heat-resilient urban planning, climate adaptation and policy in the Global South.

## 2. Background

### (a) Nighttime light (NTL) and urban productivity

NTL data have emerged as a powerful tool in socio-spatial research, offering unique insights into urban growth, economic activity and infrastructure dynamics. Owing to its direct link to human activities, NTL is widely used to characterize urban expansion and development [19,20]. Unlike daytime satellite imagery (e.g. Landsat or SPOT), NTL data provide a greater contrast between urban and rural areas, making it particularly effective for delineating the boundaries of built-up areas [21]. This characteristic has established NTL as one of the main data sets for mapping urban expansion. For example, as early as 1979, Croft demonstrated the utility of the Defense Meteorological Satellite Program - Operational Linescan System (DMSP-OLS) NTL images to map major cities in the eastern United States, highlighting their potential to represent economic growth and prosperity [22]. Since then, NTL data have become a cornerstone in urban research, offering a unique lens through which to study socio-economic and environmental phenomena.

NTL data possesses the capability to encompass a diverse array of socio-economic and environmental parameters. This richness of information proves instrumental in informing and guiding decision-making processes. Traditional metrics such as gross domestic product (GDP), population and energy consumption are often recorded at administrative levels, which can be coarse in spatial dimensions, difficult to obtain and unsuitable for granular analysis [23–25]. By contrast, NTL data provide timely, spatially explicit and consistent measurements of human activity, overcoming the delays and limitations associated with conventional data collection methods, which often suffer from lags of 1−2 years [26]. Henderson *et al.* [27] and Pinkovskiy & Sala-I-Martin [28] have shown that NTL effectively represents urban economic activity, facilitating its use in socio-economic studies.

The application of NTL data in urban studies can be broadly categorized into three streams. The first stream focuses on establishing correlations between the intensity of NTL and socio-economic variables such as GDP [29,30] and population [31]. These studies have consistently shown that the NTL radiance is a reliable indicator of economic activity, making it a valuable tool for monitoring urban productivity and development. The second stream of research emphasizes the development of statistical and ML models to explore the relationship between NTL radiance and variables of interest. Commonly used models include linear regression, panel regression, geographically weighted regression, exponential growth models and random forest [23,32–34]. More recently, deep learning techniques, such as convolutional neural networks, have been employed to predict socio-economic indicators such as GDP on finer spatial scales using NTL data as input [35]. These advances highlight the growing sophistication of NTL-based analyses and their potential to capture complex urban dynamics. The third stream of research addresses the issue of scale, which is critical for generalizing findings across different spatial contexts. Efforts have been made to bridge the gap between the training scale of models and their implementation scale, either by scaling from plot-level data to pixel-level data or by scaling from national or provincial scales to city or pixel levels [36,37]. These methodological innovations have significantly improved the applicability of NTL data in diverse urban planning and governance contexts.

### (b) Heatwave-induced productivity shifts and its measurement

Several empirical studies revealed that heatwaves can affect the economy by lowering labour productivity and increasing mortality risks [38–40]. Between 1980 and 2017, heatwaves have become prolonged, exposing urban populations to unusually high heat stress, which accounted for approximately 5% of the economic losses in Europe [41]. A prominent contributor to the exacerbation of heatwaves in cities is the UHI effect, which is sensitive to urban land use and the characteristics of the built form [1].

There is a growing literature on the attribution of heatwave events to economic and productivity losses. For example, Costa *et al.* [42] developed a comprehensive cost methodology that integrates urban climate modelling with labour productivity and economic production, revealing significant variability in the economic impacts of heat. These impacts are influenced by production characteristics, such as the elasticity of substitution between capital and labour, as well as the relative size of different economic sectors. Their findings estimate that, in the absence of adaptation measures, total economic losses during a warm year in the far future (2081−2100) could range from 0.4% of the gross value added in cities such as London, UK to up to 9.5% in cities such as Bilbao, Spain. Miller *et al.* [40] evaluated a global temperature dataset from 1979 to 2016, concluding that the potential damage to the agricultural industry could be 5 to 10 times greater than prior estimates, with manufacturing and construction also highly vulnerable to heat-related impacts.

In addition, an expanding body of literature explores regional differences in the economic impacts of urban heatwaves, highlighting concerns about environmental injustice. Callahan *et al.* [43], quantified the effects of extreme urban heat and found that human-induced increases in heatwaves (through anthropocentric emissions) have disproportionately reduced economic output in poorer tropical regions. Similarly, Garcia-Leon *et al*. [44] highlighted significant variations in economic losses from heatwaves across different spatial units, emphasizing the heterogeneity of regional impacts. These disparities are driven by the interplay of geographical and social factors, with urban green infrastructure emerging as a critical determinant. For example, a recent study showed that the uneven spatial distribution of urban green infrastructure can exacerbate economic inequality during heatwaves [2,45].

According to [1], the majority of urban heatwave research is focused on Europe, America and Asia, with almost 40% of the studies focused on Europe and America alone. This regional bias has resulted in the underrepresentation of rapidly urbanizing cities, particularly those in the Global South. Here we highlight that there is a persistent gap in the geographical coverage of heatwave impact studies, particularly in regions that undergo extreme heat events, rapid urbanization and demographic expansion. The selected subtropical case study cities based on their geographical location and shared vulnerability in diverse urban contexts that are often underrepresented in the literature on climate impact. These cities, despite differences in economic indicators or governance systems, face overlapping challenges such as infrastructural precarity, informal economies and limited adaptive capacity. These factors are not always captured through purely climatic or geographical descriptors.

Recently, a variety of econometric and ML models have been used to explore the relationship between urban heatwaves and economic losses. For example, [13] highlighted the integration of ML methods into econometric models to improve the accuracy of economic forecasts under climate projections. These advanced models enable the analysis of high-dimensional data, providing a more nuanced understanding of how urban heat stress affects economic productivity. From a dynamic modelling perspective, studies have also used time-series models such as ARX and ARMAX to examine temporal changes and forecast urban heatwaves [46,47]. However, a gap remains in the literature related to the assessment of the dynamic interactions between urban heatwaves and economic growth or activities. Bridging this gap is essential to designing better adaptive frameworks for reducing the economic consequences of heatwaves in cities, particularly in the Global South.

ML techniques have been increasingly applied to assess the vulnerability of urban heat and its economic implications. For example, [15] developed computationally efficient ML methods that enhance urban temperature estimates, which are critical for evaluating the economic benefits of heat mitigation strategies. Similarly, [48] reviewed quantitative urban models that analyse the spatial organization of economic activity within cities, offering valuable information on how UHIs influence economic outcomes. Chakraborty & Stokes [16] introduced an ML framework that adapts to city-specific NTL signatures, allowing the tracking of urban changes and providing insight into economic dynamics. This approach employs neural networks to forecast NTL patterns, allowing for the detection of deviations that signal infrastructure or economic changes.

### (c) Critique of nighttime light data

Despite the widely recognized potential of NTL data for urban mapping and measuring economic activities, its accuracy has been challenged by the blooming effect, which causes urban areas to appear larger than they actually are [49]. Addressing this issue has remained a central focus in NTL-based urban mapping research. In the early stages, researchers used the proportion of detected NTL signals relative to total cloud-free observations as an indicator to mitigate the blooming effect. The studies aimed to establish an empirical threshold for this percentage to filter out extraneous signals while maintaining consistent urban boundaries [50]. The underlying assumption was that the ‘blooming pixels’ were transient and thus had lower detection rates [27]. However, the precision of urban mapping was highly sensitive to the chosen threshold, and even within the same study area, empirical percentage thresholds varied significantly between studies [50,51]. This variability highlights the challenges in developing a universal threshold and underscores the need for more robust methods to improve the reliability of NTL-based urban mapping.

Another limitation of NTL data lies in the generalizability and scales of the models studied. Model selection can be highly dependent on the variables analysed and the specific study areas, particularly when these areas differ in socio-economic conditions, outdoor lighting regulations and population density. This variability raises concerns about the generalizability of the models, as those that perform well on a global scale may produce significant errors when applied at regional levels [52]. Furthermore, the use of different scales in the studies further complicates the generalizability of the models researched. Many studies train models at one scale and then apply them to scenarios with different spatial scales, implicitly assuming that relationships modelled at one spatial scale (e.g. national) can be directly applied to another (e.g. pixel). However, this assumption may not hold in many contexts. For example, when estimating the population using NTL data, models are typically developed based on the correlation between total NTL intensity and total population at the national level [19]. These models are then used to estimate the population at the pixel level on the basis of the NTL intensity of individual pixels. However, this approach overlooks a critical issue: most of the total intensity of NTL originates from brightly lit urban areas, while dimly lit or dark rural regions—although they host a significant proportion of the population—are often underrepresented in the models [32]. This discrepancy highlights the need for more nuanced approaches to ensure the accuracy and applicability of NTL-based models across diverse spatial scales and contexts.

Finally, several studies have concluded that relying solely on NTL data is insufficient to accurately measure GDP at subnational levels. The limitation of using NTL as the only predictor arises from its inability to represent areas without visible NTLs. Specifically, not all economic activities, such as agriculture and forestry, exhibit increased NTL emissions as they expand [53]. To address this shortcoming, researchers suggest incorporating additional data sources to improve GDP estimation. For example, [53] developed a model that integrates land cover data with NTL to estimate agricultural and non-agricultural economic growth at national and subnational levels. Similarly, [54] introduced a systematic approach for a more precise estimate of GDP by combining DMSP-OLS NTL data with enhanced vegetation index and land cover data. In addition, other parameters such as weather data, topography data and agricultural land use may be useful to complement economic analysis [55].

## 3. Data and methods

### (a) Data and preprocessing

We used the VNP46A2 dataset from NASA’s Black Marble Product Suite that records Bidirectional Reflectance Distribution Function (BRDF)-corrected NTLs from the day and night band (DNB) onboard the VIIRS instrument [56] to construct urban NTL time series over a period of 6 years (2013–2019). For each region of study, we access the time-series record from 15 February 2013 to 21 October 2019 from NASA’s Level 1 and Atmosphere Archive and Distribution System Web Interface (LAADS-DAAC). Subsequently, we follow the data preprocessing methodology of [16] to derive the gap-filled NTL time series for all pixels in urban areas corresponding to each study region, ensuring that the pixel sample contributing to the mean urban radiance of the city remains consistent. The VIIRS VNP46A2 product used in this study provides daily NTL data at a spatial resolution of 500 × 500 m. The VNP46A2’s temporal resolution is on a daily basis. We also extract the ‘mandatory quality flag’ from the VNP46A2 dataset to compute the ‘daily gap-filled fraction’ indicating the fraction of low-quality pixels in the urban area that was gap-filled using past good retrievals.

NTL data from VIIRS sensors may suffer from the blooming effect, where light from highly illuminated urban cores (e.g. city centres, industrial complexes or other high-intensity illuminations) diffuses into adjacent non-urban pixels, leading to spatial overestimation of urban extents and intensity [57,58]. To mitigate blooming, we incorporated radiance-normalized composites from the NASA Black Marble Product to reduce temporal inconsistency and sensor-induced glare, following best practices from previous studies [59]. NASA’s radiance-normalized composite includes the following strategies: (i) pixel-wise normalization, where each pixel’s radiance value is normalized to a consistent scale, typically between zero and 100, to account for varying sensor sensitivities and detector gain adjustments; (ii) temporal normalization, where data from multiple observations (e.g. daily, monthly) are combined to create a single composite image; (iii) geometric normalization is used to correct geometric distortions by projecting the data on to a consistent coordinate system using a geographic project such as WGS84; (iv) corrections for orbital and sensor effects to account for geolocation and orbital variations and sensor vignetting effects [56].

We focus on four hyperdense cities of the Global South, including Delhi (India), Guangzhou (China), Cairo (Egypt) and São Paulo (Brazil). The urban boundaries of our study areas are defined using the Joint Research Commission Global Human Settlements Functional Urban Areas dataset [60]. For all pixels in the urban areas of interest, we mask out the pixels with a gap filled NTL that matches the VNP46A2 fill value at each time step. Next, for the remaining pixels, we apply the DNB scale factor and sum the NTL radiance measurements weighted by each pixel’s area and divide by the total estimated area of all utilized pixels to derive a radiance intensity in units of nW cm^–2^ sr^–1^ (nanoWatts per square centimetre per steradian). This produces the area-weighted NTL at each time step.

**Figure 1.**
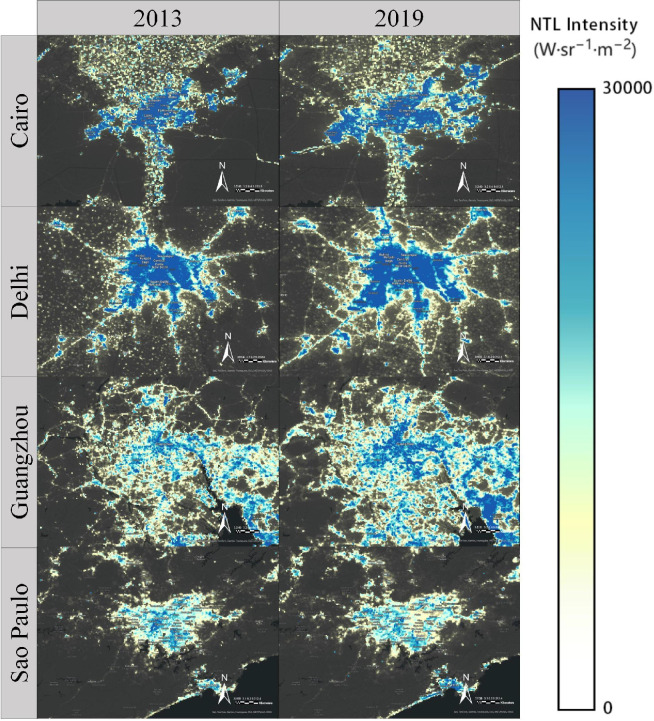
Visual representation of the annual mean NTL intensity (in nW cm^–2^ sr^–1^) for the study areas for 2013 and 2019.

Using ArcGIS Pro 3.4, customized colour ramps are implemented to distinguish between different intensity levels of artificial light emissions. High radiance values, often associated with urban centres, industrial zones and infrastructure corridors, are generally represented in warm colours (yellow to red), while lower radiance values, indicative of rural or undeveloped regions, are shown in cooler shades (blue to green) [19]. The spatial and temporal distribution of the exploratory NTL intensities is shown in figure 1. And it is apparent to visually observe the increasing trend in the NTL radiance intensity from 2013 to 2019 for the selected cities.

Historical daily weather and climate data used in this study were obtained from the Timeline Weather API [61] for the four cities. The variables queried are listed in table 1.

### (b) Conceptualization

**Figure 2.**
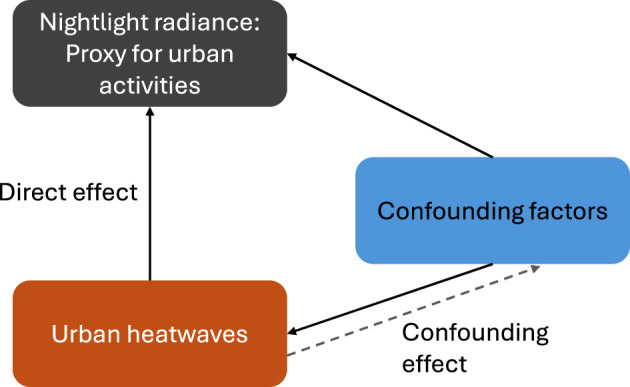
DAG representing the causal relationship between urban heatwaves and nightlight radiance as a proxy for urban activities. The dashed arrow indicate confounding effect between the variables, and the solid arrows indicate direct effect.

We conceptualized a direct relationship between heatwaves and increased economic activities at night, illustrated in figure 2 as a directed acyclic graph (DAG). We use nightlight (NTL) intensity values (in nW cm^–2^ sr^–1^) as a proxy for the representation of urban nighttime activities (after [19]), which in turn is often used as an indicator of urban economic activities [16]. In our DAG, we assume a direct impact of heatwaves on nighttime activities, denoted by increases in NTL radiance values between 2013 and 2019. In addition, we add the effects of confounding factors associated with heatwaves comprising various climate variables, lag variables and interaction terms, as listed in table 1.

Historical weather and climate data were obtained from ground-based weather stations. These station-based observations are considered highly representative for our purposes, as they provide accurate point-based measurements that can be spatially matched to urban areas using averaging or buffering techniques. Given that both VIIRS radiance values and climate indicators were aggregated at the city level, the station data offer a meaningful basis for comparison with the weighted-average radiance intensity across each urban extent. The spatial locations of the weather stations are checked and ensured to be in the central urban regions.

### (c) Methodology

In empirical research, traditional parametric methods, such as ordinary least squares (OLS), fixed effects models and instrumental variables, are widely used to estimate causal relationships. These methods typically rely on strong functional form assumptions (e.g. linear relationships) and a relatively low-dimensional set of predictors. Although these assumptions can simplify interpretation, they become problematic when dealing with highly complex nonlinear processes or a large number of confounders [62–66]. Many methods assume a ‘sparse’ structure, implying that only a small number of covariates significantly affect the outcome, even if a large number are available [67].

A key objective in much of this causal inference literature is average treatment effects (ATE) under the unconfoundedness assumption. Under this assumption, ATE can be identified by conditioning on observed covariates, ensuring that treatment assignment is independent of potential outcomes given these controls. In traditional econometrics, researchers handle unconfoundedness primarily through explicit modelling and adjustment techniques [68,69]. However, these approaches often struggle with high-dimensional confounders and complex functional relationships. In our context of evaluating the ATE of heatwaves on NTL emissions, the sheer variety of climatic, infrastructural and socio-economic factors can render a purely parametric specification vulnerable to misspecification potentially biasing causal estimates.

Recent advances in ML-based causal inference methods, especially double machine learning (DML), provide a flexible, non-parametric approach to modelling confounders while preserving valid inference for causal parameters [70–73]. In this study, we use this approach with the detailed methodological specifications presented below.

To investigate the temporal predictive relationship between climate variables and NTL intensity, we also used Granger causality tests. Although our primary causal inference relies on the DML framework, the Granger test serves a complementary role by assessing whether past values of climate indicators hold predictive power for current variations in radiance intensity. We do not interpret Granger causality as establishing definitive causal directionality. Rather, it provides empirical support for temporal precedence, which is useful in informing the structure of the DML model and interpreting potential mediation dynamics. In the context of this study, the test evaluates whether prior changes in air temperature, a proxy for heatwave events, help predict subsequent changes in NTL radiance, which serves as a proxy for urban activity intensity. Given the daily frequency of the data and the need to capture short-term behavioural responses to temperature fluctuations, we used a lag duration of 2 days, a range commonly applied in urban temporal studies and aligned with the lag structures used in our robustness checks (see electronic supplementary material, A6–A7). The results, presented in electronic supplementary material, table A3, confirm the statistically significant Granger causality (*p* < 0.01) in all four cities studied, indicating that heatwaves have a predictive effect on NTL intensity. This strengthens our argument that heat-induced behavioural changes, such as shifts in work hours, energy use and economic activity at night, are observable in high-frequency urban light data and can be systematically assessed through causality frameworks.

#### (i) Modelling approach

First, we start our analysis by defining the conditions for heatwaves using a two-stage approach.

*Hot day identification.* For each city c, daily average temperatures $T_{c,t}$ were compared with a percentage-based threshold. The daily average temperature was used because it reflects the cumulative thermal load that drives such behavioural adaptations more effectively than a single-point minimum or maximum measure. Using daily averages, we align our identification of hot days with the broader temporal scope of heat exposure that shapes economic decisions, especially in rapidly urbanizing regions. Let $\tau_{c,0.95}$ denote the 95th percentile of the city-specific distribution of the daily average temperature $T_{c,t}$ calculated over the warm seasons of 2013−2019. The hot day indicator is then defined as


(3.1) $$\begin{array}{r} I_{c,t}=\left\{ \begin{array}{ll} 1, & \text{if }T_{c,t}\geq \tau_{c,0.95},\\ 0, & \text{otherwise}. \end{array}\right. \end{array}$$

*Heatwave identification.* Heatwaves in this study were defined using a relative percentile-based threshold aligned with established heat-health research studies [74]. Specifically, we identified a heatwave event when the daily mean temperature exceeded the local 95th percentile of the warm season $T_{mean}$ (2013−2019) for at least two consecutive days. We also examine a more stringent definition of at least three consecutive exceedances, matching the World Health Organization heat-health guidelines that recommend heatwave durations of ‘2 to 3 days or longer’ [75]. Thus, both 2- and 3-day criteria were considered for robust event identification within each city. This 95th percentile on two to three consecutive days mirrors the benchmark used in leading multi-city heat-mortality studies. [76–79].

First, for each city c we flag a hot day $I_{c,t}=1$ whenever $T_{\mathrm{mean},c,t}\geq \tau_{c,0.95}$ (95th percentile threshold).

Second, we aggregate those hot days across consecutive periods of length d to obtain


(3.2) $$\begin{array}{r} {\text{HW}}_{c,t}^{\left(p,d\right)}=\left\{ \begin{array}{ll} 1, & \text{if }\sum_{k=0}^{d-1}I_{c,t-k}\geq d,\\ 0, & \text{otherwise}. \end{array}\right. \end{array}$$

Thus, $HW_{c,t}^{\left(p,d\right)}=1$ whenever the temperature in the city c exceeds its 95th percentile threshold for d consecutive days. We apply this rolling-sum logic for different durations d ∈ {2,3,4} to assess how longer heatwaves affect outcomes differently than shorter ones.

This two-stage approach, first identifying days at or above the percentile threshold and then aggregating consecutive hot days, provides flexibility in capturing varying intensities and durations of heat stress in different climate regimes.

#### (ii) Double machine learning approach

Now, we estimate the effect of these heatwave events on NTL using a debiased DML framework. For each percentile-duration pair (p,d), we define $Y_{c,t}$ as the NTL ($\log(NTL_{t})$) intensity (our outcome of interest in radiance), ${\text{HW}}_{c,t}^{\left(p,d\right)}$ as binary treatment (heatwave indicator) and X as a set of covariates. Fixing city c, consider two otherwise identical days, one that falls within a qualifying heatwave spell of length d and one that does not. We then define $\theta^{\left(p,d\right)}$ as the average causal effect within the city of a (p,d) heatwave spell on NTL:


$$\theta^{\left(p,d\right)}\;=\;E\;[Y_{c,t}(1)-Y_{c,t}(0)],$$

where $Y_{c,t}(1)$ is log(NTL) for a city c on day t when the day lies within a qualifying heatwave spell, and $Y_{c,t}(0)$ is log(NTL) for the same city day under non-heatwave conditions. Because the expectation is taken in a fixed city, $\theta^{\left(p,d\right)}$ captures variation solely within each city and does not involve cross-city comparisons. Our analysis is purely within the city and does not rely on differences across cities.

Following [17], we consider the partially linear regression model:^1^


(3.3) $$Y_{c,t}=\theta^{\left(p,d\right)}\cdot {\text{HW}}_{c,t}^{\left(p,d\right)}+g(X_{c,t})+\epsilon_{c,t},E[\varepsilon_{c,t}|X_{c,t}]\;=\;0\;,$$

(3.4) $${\text{HW}}_{c,t}^{\left(p,d\right)}=m(X_{c,t})+\nu_{c,t},E[\nu_{c,t}|X_{c,t}]\;=\;0\;,$$

where g(.) and m(.) are unknown nuisance functions, $\epsilon_{c,t}$ and $v_{c,t}$ the error terms, and $\theta^{\left(p,d\right)}$ denote the causal ATE of experiencing a heatwave at threshold p and duration d. The optimal duration was chosen based on the most significant results. The theta term shows this and its physical significance.

The mechanistic process behind the causal interpretation of ATE $\theta^{\left(p,d\right)}$ derived from our partial linear regression model is based on the foundational work by [17] and [69], where the causal interpretation hinges on three core assumptions for causal interpretation: (i) conditional independence (unconfoundedness), where confounders $X_{c,t}$ must satisfy $Y_{c,t}(1),\;Y_{c,t}(0)\;⊥\;⊥\;{HW}_{c,t}^{\left(p,d\right)}|X_{c,t}$ . This implies that, conditional on covariates $X_{c,t}$, the treatment assignment (heatwave occurrence) is independent of potential outcomes. In our setting, $X_{c,t}$ includes time-varying confounders (e.g. day of week, seasonality, weather controls) and city-specific trends that may jointly influence both heatwaves and NTL. The DML framework flexibly accounts for these confounders through the nuisance functions g(.) and m(.). Next, we accounted for (ii) overlaps: $0<\mathbb{P}({HW}_{c,t}^{\left(p,d\right)}=1|X_{c,t})<1$, which ensure that there is a non-zero probability of both treated and untreated observations for all covariates. This assumption holds true in our case, as the occurrences of heatwaves in cities and time were variable. Finally, (iii) DML’s partial linear model isolates $\theta^{\left(p,d\right)}$ through double robust orthogonalization. We used ML to estimate g(.) (outcome model) and m(.) (propensity model) without imposing restrictive parametric assumptions, thus avoiding misspecification bias. In addition, by splitting the data into folds (cross-fitting) we avoid using the same observations for both training and final estimation of nuisance functions, thereby reducing overfitting. Last, we leveraged the Frisch–Waugh–Lovell theorem to partial out the effect of $X_{c,t}$ through orthogonalization. The final ATE estimate is obtained by regressing the residualized outcome on the residualized treatment, which isolates the variation independently of $X_{c,t}$ (expanded in the following sections).

*Nuisance function estimation.* To operationalize DML, we must estimate the two ’nuisance’ components: (i) the outcome regression function g(.) and (ii) the treatment assignment function m(.).

(i) *Outcome model* (g), estimated using *lasso regression*:

(3.5) $$\begin{array}{lcr} & \hat{g}(X_{c,t})=\arg\;\min_{g}\left\{\frac{1}{n}\sum_{c,t}{\left(Y_{c,t}-g(X_{c,t})\right)}^{2}+\lambda \sum_{j=1}^{J}|\beta_{j}|\right\}, & \end{array}$$

where λ is the regularization hyperparameter (often chosen via cross-validation), $\beta_{j}$ are the regression coefficients on each feature j and n is the total number of observations. By shrinking certain $\beta_{j}$ towards zero, lasso simultaneously performs variable selection and regularized estimation, allowing g(.) to capture high-dimensional or potentially sparse relationships.

(ii) *Treatment model* (m), estimated using *random forest*:

(3.6) $$\begin{array}{lcr} & \hat{m}(X_{c,t})=\frac{1}{B}\sum_{b=1}^{B}{\text{Tree}}_{b}(X_{c,t}), & \end{array}$$

where each $Tree_{b}(\cdot )$ is grown on a bootstrap sample of the data (with random feature splits), and B is the total number of trees in the ensemble. This approach allows for nonlinear and interaction effects among covariates when predicting ${\text{HW}}_{c,t}^{\left(p,d\right)}.$

*Orthogonalization and cross-fitting.* DML proposed by [17] leverages orthogonalized cross-fitting, making it less susceptible to overfitting and model misspecification than naive ML regressions. Since semi-parametric methods rely on model quality for the estimation of nuisance parameters, critics point out that they can be sensitive to the inclusion of irrelevant or endogenous variables, potentially inflating variance and biasing estimates [80]. Similarly, the performance of DML is highly dependent on the correct specification of the ML models used for the estimation of nuisance parameters [17]. Fuhr J. [18] highlights that while DML can adjust for various nonlinear confounding relationships, it still critically depends on standard assumptions about causal structure and identification. Therefore, careful consideration is necessary when selecting control variables and specifying models within the DML framework to ensure valid causal inference.

Cross-fitting is central to DML because it ensures that no observation is used to train the nuisance functions that are then applied to the same observation in the final stage. This protects against overfitting and yields more reliable standard errors. Concretely:

(i) *Data splitting.* Divide the sample into K folds (*i.e*. K=10 in our baseline setting). Denote each fold by $I_{k}$.
(ii) *Training and prediction.* For each fold k:
  — Train the nuisance models ${\hat{g}}^{\left(k\right)}$and ${\hat{m}}^{\left(k\right)}$on all folds except $I_{k}$.
  — Predict on the held-out fold $I_{k}$, obtaining residuals:

(3.7) $$\begin{array}{lcr} & {\tilde{Y}}_{c,t}^{\left(k\right)}=Y_{c,t}-{\hat{g}}^{\left(k\right)}(X_{c,t}), & \end{array}$$

(3.8) $$\begin{array}{lcr} & {\tilde{D}}_{c,t}^{\left(k\right)}={HW}_{c,t}^{\left(p,d\right)}-{\hat{m}}^{\left(k\right)}(X_{c,t}). & \end{array}$$

  Here ${\tilde{Y}}_{c,t}^{\left(k\right)}$ is the outcome partialed out by the predicted outcome model, and ${\tilde{D}}_{c,t}^{\left(k\right)}$ is the treatment partialed out by the predicted treatment model.

(iii) *Final estimation of* **$\theta^{\left(p,d\right)}$**. After obtaining all residuals ${\tilde{Y}}_{c,t}^{\left(k\right)}$ and ${\tilde{D}}_{c,t}^{\left(k\right)}$ from each fold, DML stacks them together and runs a simple linear regression (or direct method-of-moments formula):

(3.9) $$\begin{array}{lcr} & {\hat{\theta }}^{\left(p,d\right)}={\left(\sum_{c,t}{\tilde{D}}_{c,t}^{2}\right)}^{-1}\left(\sum_{c,t}{\tilde{D}}_{c,t}{\tilde{Y}}_{c,t}\right). & \end{array}$$

This step effectively regresses the partialed-out outcome ${\tilde{Y}}_{c,t}^{\left(k\right)}$ to the partialed-out treatment ${\tilde{D}}_{c,t}^{\left(k\right)}$. Because the nuisance models were trained on different folds than those used for the final regression, the resulting estimator ${\hat{\theta }}^{\left(p,d\right)}$ is orthogonal (*i.e. ‘doubly robust’*) to small errors in $\hat{g}$ and $\hat{m}$.

*Estimation approach by lasso and random forest.* Lasso is a penalized regression method designed to handle highly dimensional or potentially sparse data. Lasso and tree-based methods, such as random forests and boosted trees, can perform variable selection by default. That is, they will only make use of features that are predictive of the outcome, which enables them to work in high-dimensional settings with many variables or transformations. In our study, we implement lasso with the glmnet package (v 4.1) and random forest with the ranger package (v 0.7.0) with default settings in R.

*Model assumptions.* We follow the specifications of [73] to obtain a reliable treatment estimate from observational data using causal inference methods. Several assumptions must be met, most importantly unconfoundedness, ignorability, positivity and the stable unit treatment value assumption (SUTVA). Unconfoundedness asserts that covariates encapsulate all influencing factors on both treatment and outcome, leaving no confounders unaccounted for, thus removing confounding bias.

Ignorability asserts that, when conditioned on observed covariates, the assignment of treatment is autonomous of potential outcomes. As such, units could be swapped between the treatment group (presence of a heatwave event) and control group (absence of heatwave event) without affecting the experiment (exchangeability). Incorporating inappropriate controls, such as variables that can influence heatwave estimations like the UHI effect or the specific definition of heatwaves used by the region, breaches the assumption and skews the effect estimate. Therefore, in our study, we use cooling degree days (CDD) to account for the region-specific definition effect.

CDD is used as a standardized indicator to facilitate inter-city comparisons of the effects of heatwaves on energy use. However, temperature itself, while a direct measure of thermal conditions, is not a static or controlled variable in our analysis. The UHI effect, which is a key component of the relevance of CDD, varies significantly between cities owing to factors such as geography, urban morphology and local climatic variations. Temperature data alone may not fully account for regional differences in climate patterns, such as UHI effects or variations in seasonal temperature ranges. Hence, by incorporating CDD as a controlled variable, we ensure that we are including a standardized measure of thermal load. For example, CDD is a standardized measure that quantifies the number of days with temperatures above a threshold value (usually 18°C), adjusted for the average temperature. This standardized approach allows for more accurate comparisons in cities with different climatic conditions [81]. Furthermore, by using CDD as a confounding variable, the study can account for potential biases resulting from differences in the definition of heatwaves between regions.

Furthermore, by incorporating CDD as a confounding variable, we can better control for climate-driven variability in NTL activities. This helps reduce the risk of spurious associations and ensures a more accurate estimate of the relationship between NTL and heatwaves.

Positivity states that, across the range of covariates, there must be a positive probability that observations are present in the treatment group. In other words, there should be a reasonable overlap between the covariate distribution of the treatment and control groups. Last, SUTVA states that assigning treatment to one unit does not affect the potential outcomes of other units. It assumes that there are no interference or spillover effects between units. It also states that there is only one consistent version of treatment. In our case, the climatic variables used are physical phenomena and do not interfere with NTL activities. We introduce lag variables to capture these spillover effects (see table 1). For the moment, we assume that these assumptions hold sufficiently and discuss potential violations and implications for the robustness of our results and the applicability of the model under discussion.

#### (iii) Evaluation

To facilitate the interpretation of the treatment effect, we report it as a log of average NTL radiance compared with heatwave days across each city. As our unit of analysis is the heatwave events, we report the absolute individual treatment effect (ATE) in the direction of more nighttime activities owing to heatwaves, i.e. higher heatwave events and greater nightlight radiance. Here, ATE gives an indicator of how much the existing difference in heatwave events affects nighttime activities in Delhi, Guangzhou, Cairo and São Paulo.

To examine the robustness of our results, we performed the following experiments: (i) We assess the existence of threshold effects and other nonlinearities of the influence of the heatwave on NTL in the study areas using an XGBoost model and discuss their implications for our DML approach. (ii) We performed a sensitivity analysis to assess the variations in the NTL values before and after the heatwave analysis. Here, we considered the start of the heatwave event as the baseline (0th day), and 2 days before and 5 days after this baseline event. Furthermore, we address the low explanatory power and the resulting high uncertainty of treatment effect estimates by performing lag event analysis by 1, 2 and 3 days, respectively, across temperature, humidity and CDD confounders (see table 1). Detailed results of the robustness check are presented in electronic supplementary material, A5–A6.

## 4. Results

Before reporting the DML results, we conducted the Granger causality test to explore causal directionality to explore whether there is a relationship between daily air temperature and NTL activities in the four cities. We used Akaike information criteria and Bayesian information criteria to determine the model fit with a lag of two and three (following the lag variables listed in table 1). We find statistically significant associations (99% condifidene interval) between air temperature and NTL activities in all cities. This suggests that elevated air temperatures commonly observed during heatwave episodes have a direct relationship to higher NTL radiance values, a proxy for increased nighttime activities. In addition, we performed the augmented Dickey–Fuller test for all cities, which confirmed stationarity for NTL and air temperature time-series data in the study areas. Extended descriptive results are presented in electronic supplementary material, A1–A3.

### (a) Threshold selection

Table 2 lists a summary of the sample size, city‐specific 95th percentile thresholds, and the count of multi-day hot spell episodes at each location over the time period (2013–2019). All four cities exhibit approximately 2450−2540 valid warm season days. However, their absolute threshold levels differ from 27°C in São Paulo to 37°C in Delhi, reflecting distinct baseline climate. Once we impose the ‘equal to or greater than’ 95th percentile $T_{\text{mean}}$ for d*-days* rule, episode counts vary sharply. São Paulo and Cairo see only 22−24 2‐day spells and even fewer 3-day events (715), while Guangzhou, with a more moderate threshold, experiences 64 2-day and 34 3-day hot spells. The drop in spell counts as d increases highlights why we focus on both 2- and 3-day definitions, ensuring that we capture enough episodes for robust inference while still isolating truly extreme, locally rare heat events.

**Table 2.** Sample size, temperature threshold and hot-spell days.

	valid	95th percentile	hot-spell days ( ≥d consecutive hot days)		
city	days	$T_{\text{mean}}$ (°C)	d≥2	d≥3	d≥4
São Paulo	2469	27.0	24	15	11
Cairo	2540	32.5	22	7	3
Delhi	2465	37.1	24	13	8
Guangzhou	2451	31.1	64	34	18

### (b) Direct relationships

Table 3 shows that Guangzhou, Cairo and São Paulo all register a statistically significant rise in NTL radiance when a heatwave lasts at least 2 days, indicating a rise in nighttime activities. However, when we extend the heatwave definition beyond the 2-day threshold, the incremental increase in the NTL values is reduced. For example, the increase in NTL in Guangzhou falls from approximately 34% to 24%, with similar drop-offs in Cairo (from 52% to 32%) and São Paulo (29% to 19%).

**Figure 3.**
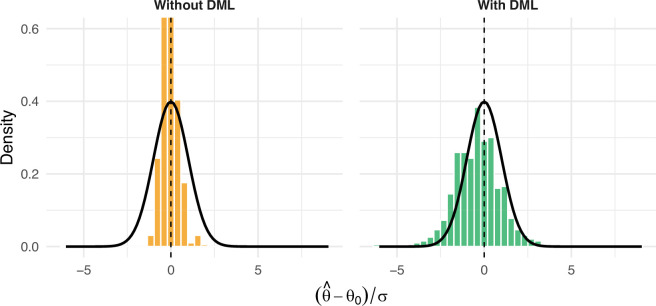
Bootstrap sampling‐distribution of the standardized heatwave coefficient from an OLS specification (left) and the cross-fit DML estimator (right).

**Table 3.** Estimated NTL response during multi-day heat spells. Reported numbers are the percentage change in NTL radiance relative to non–heatwave days for spells of ≥2 consecutive hot days (*d* = 2) and ≥3 consecutive hot days (*d* = 3); *p*-values test the null of no effect; asterisks indicate rejection at the conventional 95% confidence threshold (*<0.05, **<0.01, ***<0.001).

	*d* = 2 (≥2 days)		*d* = 3 (≥3 days)	
city	effect	*p*‐value	effect	*p*‐value
Guangzhou	**+34%**	0.00895**	**+24%**	0.00175 **
Cairo	**+52%**	0.0456*	**+32%**	0.037 *
São Paulo	**+29%**	8.86×10^−5^***	**+19%**	0.0285*
Delhi	**+0.027%**	*	**−17%**	0.00516 **

Three-day heatwave spells occur infrequently, so their effects are drawn from fewer observations, limiting statistical power to detect large NTL changes. This event rarity factor helps explain why the NTL increase drops off for 3-day spells, even as the first two hot nights showed sharp rises. Delhi diverges from the other cities (see table 3). At 95% relative threshold 2-day heatwaves have no detectable effect on NTL values, while longer spells (d≥3) reduce radiance by 17%.

As a sensitivity analysis, we applied [82] impulse response function-based local projection to each of our four cities to trace the day-by-day response of NTL radiance before and after heatwave events. Guangzhou, Cairo and São Paulo show a similar post-event trend (see figure 3). Once a heatwave occurs, the NTL intensity drifts upward for two or three nights, reaches a short-lived peak, and then fades back towards its usual level. This convex profile points to a short-lived possible reallocation of activities.

As shown in figure 3*b*, Delhi’s response remains relatively unchanged after the heatwave event, drifting slightly downward rather than showing the pronounced surge observed elsewhere. Deriving a causal implication on why this pattern is unique to Delhi is beyond the scope of this study.

In addition, to demonstrate the value addition of using DML as a superior multi-variate statistical estimator, we benchmark our empirical set-up against a conventional OLS specification that uses the same controls but omits the cross-fit and orthogonalization steps of DML; see figure 3.

## 5. Discussion

**Figure 4.**
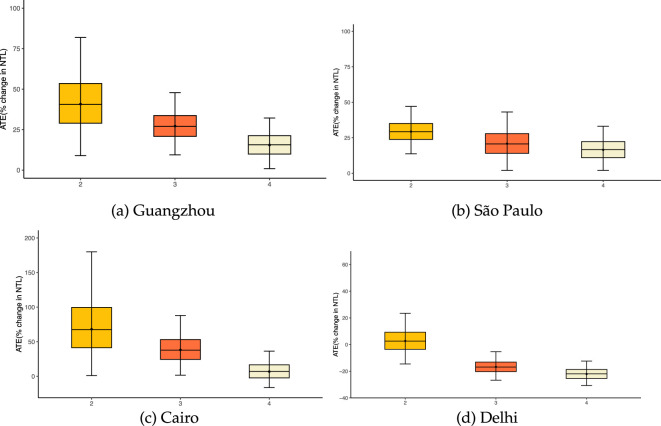
Impact of multi-day heatwaves on NTL intensity in four megacities. Each panel shows the estimated ATE on NTL radiance, expressed as percentage change, for spells of two, three and four consecutive hot days (yellow, orange and cream boxes, respectively). Coloured boxes depict the inter-quartile range of the simulated sampling distribution (whiskers suppressed); the thin black line shows the 95% Wald confidence interval and the dot marks the coefficient estimate. Positive values imply brighter lights during heatwaves relative to non-heatwave days. Axes are scaled separately to emphasize within-city variation.

**Figure 5.**
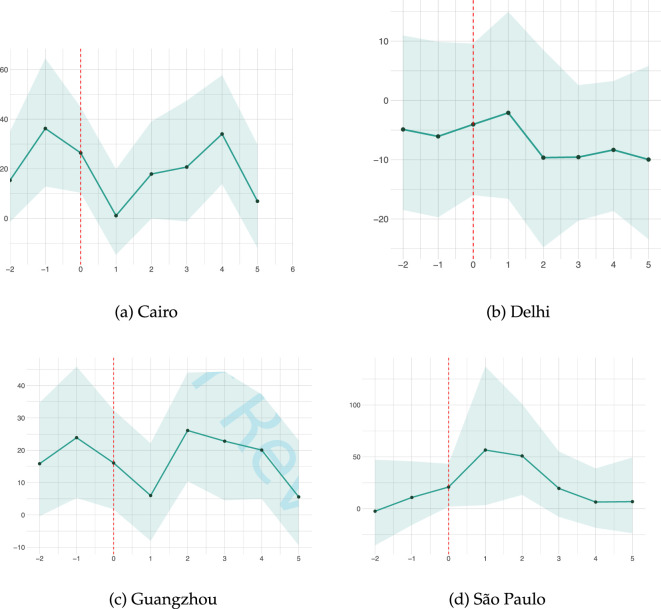
Sensitivity analysis of heat‐wave impacts on NTL radiance in the four cities. The horizontal axis gives day h relative to the first heatwave day (−2 = 2 days before, +5= 5 heatwaves after), while the vertical axis plots the estimated percentage change in average NTL radiance. Solid markers connected by a teal polyline show the point estimate at each horizon; the vertical red dashed line marks h=0. Shaded bands represent 95% confidence intervals.

In this study, we investigated the relationship between heatwave events and NTL radiance values from 2013 to 2019 in four hyperdense cities in the Global South using a DML framework. We find a statistically significant direct link between heatwaves and the elevated intensity of NTL in Delhi, Guangzhou, Cairo and São Paulo (see figure 4 and table 3). Furthermore, a sensitivity analysis was performed to assess the robustness of these findings by evaluating changes in NTL intensity before and after the heatwave (see figure 5). This sensitivity analysis reveals consistent and statistically significant heatwave-NTL response patterns in the study areas (except Delhi) for a 2-day heatwave spell. We did not find such a pattern for a 3-day heatwave spell as a result of fewer observations.

Our results support the hypothesis that as extreme temperatures increase, nighttime activities increase (proxied through NTL values). This signal calls for adaptive policies and urban planning interventions that can accommodate such structural shifts without exacerbating energy demand, public health and socio-economic inequalities. For example, investing in heat-resilient infrastructure that includes built environments designed to prevent direct heat exposure and support adaptation through features such as urban greening, shaded public spaces and accessible cooling mechanisms. Similarly, limiting human exposure to heat during peak heat hours through rescheduling economic activities, thereby limiting health risks and maintaining productivity. Effective and early heat warning systems can provide timely and localized alerts that inform individuals and businesses when to adapt or change activities in response to forecast heat extremes.

Urban planning instruments can be useful for adapting to heatwaves. For example, a crucial policy intervention involves the expansion of urban green infrastructure, as research has consistently demonstrated that vegetation plays a significant role in reducing the effect of UHIs. Trees, green roofs and vertical gardens can lower surface and ambient temperatures, particularly in dense urban areas where built environments retain excessive heat [1,2]. Beyond green infrastructure, urban zoning and building codes must be upgraded to allow adaptation to heatwaves. For example, cities can mitigate the effect of UHIs by implementing climate-adaptive zoning policies that require the use of high-albedo materials, cool roofs and reflective pavements in new developments [1]. In addition, zoning regulations in such areas could be revised to incorporate ventilation corridors, open spaces that facilitate natural airflow, and reduce localized heat buildup.

The findings of our study indicate an immediate imperative to reconsider adaptation strategies in urban environments with regard to heatwaves in crowded and rapidly urbanized cities, with nighttime illumination serving as a potentially significant indicator in the formulation of these strategies. From a methodological perspective, this study advances the current scholarly discourse regarding the application of causal ML techniques in sustainable environmental design.

## 6. Limitations and future work

This study has certain limitations related to our conceptualization of the direct impact of heatwaves on NTL intensities in study areas between 2013 and 2019. Although the validity of NTL as a proxy for urban economic activity is well-established in the literature, it is usually affected by multiple and complex confounding factors, stemming from the socio-demographic and cultural fabric of urban spaces, which are difficult to objectively specify in a data-driven model like DML. Similarly, different cities have different NTL regulations which also affect their measurement from space. Furthermore, remote sensing data used for NTL analysis may be subject to technical limitations, such as sensor resolution, atmospheric interference and temporal inconsistencies in satellite data collection. These factors can affect the accuracy of NTL measurements, potentially leading to discrepancies in observed trends. Future research could benefit from integrating complementary data sources, such as high-resolution socio-economic indicators or localized surveys, to improve the robustness of the findings. Using data sets from more recent satellites such as Jilin-1 may be more advantageous. This is because the resolution would be around 130 m compared with the 500 m for the VNP46A2. Future research may further study the usefulness of different satellite data.

Furthermore, in conceptualizing how the urban UHI interacts with heatwaves, we adopted a simplified aggregated definition. The interactions between UHI and heatwaves can be complex, especially within intricate urban structures such as density, diversity and mixed land use areas in cities. The disentanglement of these interaction effects remains an unresolved question and is a future research avenue. In addition, we did not consider confounding factors resulting from urban morphologies in our study regions, such as the integration of shopping centres with residential areas, which may affect the intensities of the NTL. Furthermore, dimly lit or dark rural regions—although they host a significant proportion of the population—are often underrepresented in the models [32]. But since our focus on this paper is urban activity and economic activity, our key signals came from high-radiance intensities (brightly lit) urban cores (industrial zones, commercial districts, city centres). In that sense, low-intensity rural lights that may be negligible are unlikely to capture the city-centric patterns we care about. Hence, our scope was limited to urban centres.

Ongoing advances in DML frameworks and the rapid growth of causal ML underscore the potential for confounding errors in our models despite rigorous robustness checks and evaluations. Cross-validation using newer model alternatives such as physics-based ML can guide future extensions of this work.

## Data Availability

All datasets used in this study are available in the public domain: (i) Nightlight data using the VNP46A2 dataset from NASA’s Black Marble Product Suite and (ii) Timeline Weather API for daily climate data. The codes used in this study are available at https://github.com/SimonRogersHan/Climate-Data. Supplementary material is available online [83].

## Footnotes

^1^
DML allows the use of variety of different predictive or ML methods for the treatment and outcome model [17]. Depending on the empirical setting, one might use different ML algorithms (e.g. gradient boosting, neural networks) in place of lasso or random forest. The key requirement is that each nuisance model $\hat{g}(\cdot )$ and $\hat{m}(\cdot )$ should be sufficiently flexible to capture confounding relationships without imposing overly restrictive functional forms.
